# Maternal obesity induced metabolic disorders in offspring and myeloid reprogramming by epigenetic regulation

**DOI:** 10.3389/fendo.2023.1256075

**Published:** 2024-01-16

**Authors:** Joo Young Kweon, Hyeonji Mun, Myeong Ryeol Choi, Hong Seok Kim, Yong Joo Ahn

**Affiliations:** ^1^ Medical Science and Engineering, Graduate School of Convergence Science and Technology, Pohang University of Science and Technology, Pohang, Republic of Korea; ^2^ Department of Molecular Medicine, College of Medicine, Inha University, Incheon, Republic of Korea; ^3^ Department IT Convergence, Pohang University of Science and Technology, Pohang, Republic of Korea

**Keywords:** maternal obesity, diabetes, reprogramming, fetal development, epigenetic regulation

## Abstract

Maternal obesity and gestational diabetes are associated with childhood obesity and increased cardiovascular risk. In this review, we will discuss and summarize extensive clinical and experimental studies that metabolically imbalanced environment exposure in early life plays a critical role in influencing later susceptibility to chronic inflammatory diseases and metabolic syndrome. The effect of maternal obesity and metabolic disorders, including gestational diabetes cause Large-for-gestational-age (LGA) children to link future development of adverse health issues such as obesity, atherosclerosis, hypertension, and non-alcoholic fatty liver disease by immune reprogramming to adverse micro-environment. This review also addresses intrauterine environment-driven myeloid reprogramming by epigenetic regulations and the epigenetic markers as an underlying mechanism. This will facilitate future investigations regarding maternal-to-fetal immune regulation and the epigenetic mechanisms of obesity and cardiovascular diseases.

## Introduction

**Figure 1:**
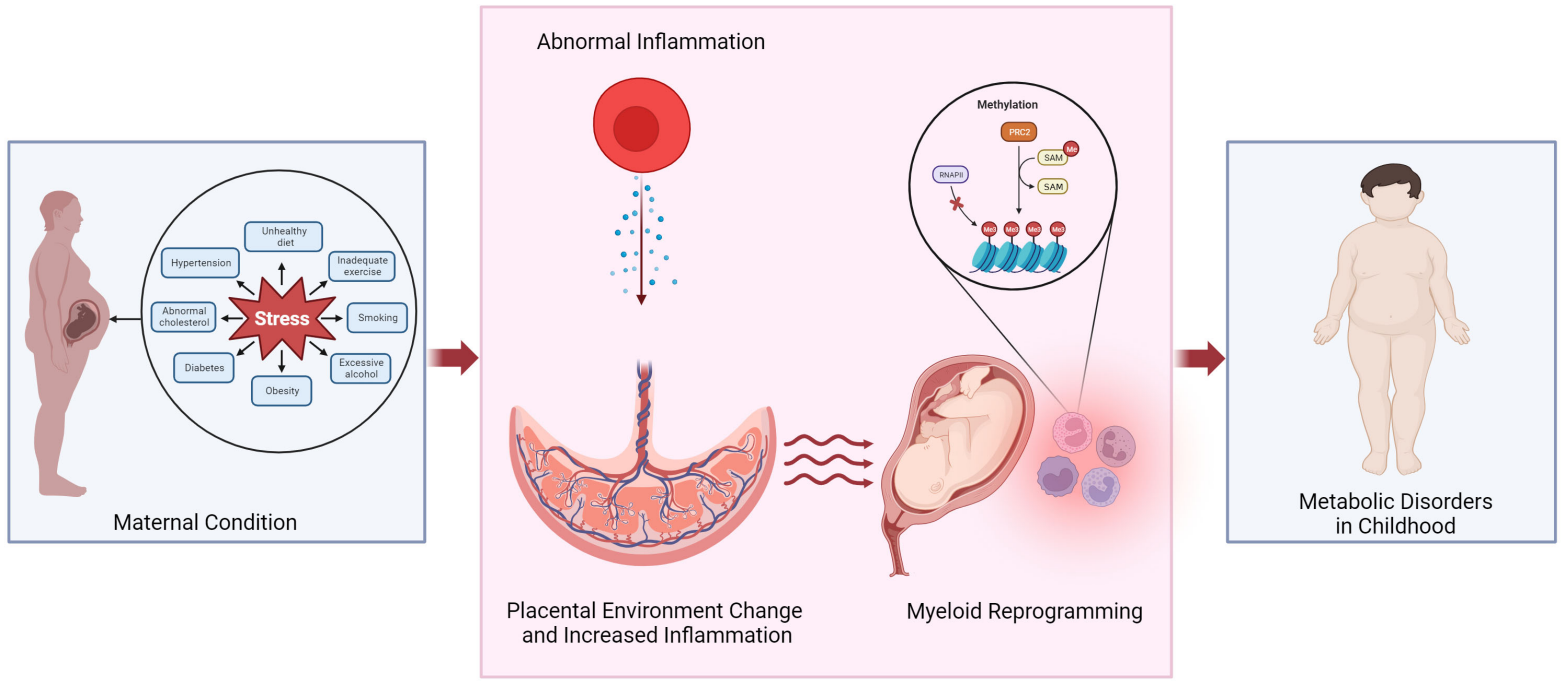
Potential mechanism linking altered placental environment and increased risk of metabolic disorders in childhood following exposure to abnormal maternal inflammation. We propose that obesity or pregnancy complications can trigger an inflammatory state within the placenta, leading to the reprogramming of myeloid cells and in the fetal cord blood. Epigenetic reprogramming could play a role in mediating the development of metabolic diseases in the offspring. PRC2, polycomb repressive complex 2; RNAPII, RNA polymerase II; SAM, S-Adenosyl methionine.

Maternal obesity and gestational diabetes have been linked to increased risk of childhood obesity and cardiovascular disease. The prevalence of obesity in women of childbearing age has been increasing, leading to various complications that can also impact both the mothers and the fetus. According to the report by the Centers for Disease Control and Prevention in 2022, 2% to 10% of pregnant women in the United States experience gestational diabetes (1). Maternal obesity, both before and during pregnancy, contributes to complications during pregnancy, such as gestational diabetes and delivery of large-for-gestational-age babies. Additional evidence supports that the maternal environment’s developmental reprogramming influences fetal and infant development, altering the risk profile for disease later in life (**Figure 1**). This review highlights the study of diseases caused by maternal origin to the fetus and affects childhood.

## Maternal underlying conditions and the impact on fetal health conditions

Maternal obesity has known as a risk factor for cardiometabolic diseases. A study by Gaillard et al. investigated the relationship between maternal and paternal obesity and childhood health outcomes. Maternal obesity was strongly correlated with childhood total body and abdominal fat mass, as well as increased risk factors for cardiometabolic diseases. Childhood obesity and cardiometabolic disease risks were assessed during the different periods of the pregnancy. The findings indicated that higher weight gain in early pregnancy was associated with increased childhood BMI and fat mass. However, weight gain in mid-to-late pregnancy did not show the same association. It is important to note that the study did not specifically explore the mechanisms underlying the relationship between maternal obesity and childhood overweight. Therefore, the precise pathways through which maternal obesity influences childhood weight were not investigated in this study (2, 3).

A study conducted by Catalano et al. examined perinatal risk factors for childhood obesity. They found that maternal pregravid obesity, as determined by BMI, was the most influential perinatal predictor of childhood obesity, in contrast to maternal glucose homeostasis or weight gain during pregnancy. In a prospective study, women with impaired glucose tolerance or gestational diabetes, along with their children, were evaluated at birth and at age. Various data points, including obstetric information, parental anthropometry, and neonatal body composition, were collected and assessed at birth, and diet and activity were assessed at follow-up. Interestingly, in this study, there was no correlation between birth weight and the child’s weight at the follow-up period. However, there was a significant correlation between body fat percentage at birth and follow-up. This suggests that the amount of body fat a newborn possesses may have implications for their weight and body composition later in life. The strength of this study is that it was conducted in a longitudinal cohort that was prospectively studied for more than 18 years. This study highlights the importance of maternal pregravid BMI as a significant factor in fetal obesity. However, a limitation is the relatively small number of subjects (4).

Children born to obese mothers have increased risk of respiratory diseases. Maternal pre-pregnancy obesity was associated with an increased risk of frequent wheezing in children, although no significant association was found with infrequent wheezing (5). In another study conducted by Rajappan et al. focused on the relationship between maternal pre-pregnancy body mass index (BMI), weight gain during pregnancy, and the occurrence of wheezing, and respiratory symptoms in children before a year of age. The finding revealed that higher maternal BMI, regardless of maternal weight gain during pregnancy and paternal BMI, was associated with an increased risk of bronchial hyperreactivity and lower respiratory tract infections in children (6). These observations further contribute to the growing body of evidence supporting the potential impact of *in-utero* exposure on respiratory disease-related phenotypes in the context of maternal obesity.

During the maturation of immunologic functions in the early stages of childbirth, a number of bacterial and viral infections typically emerge. Maternal immunological disturbances have been recognized as significant contributors to the development of fetal immunity, although the underlying processes are not yet fully understood. In this respect, maturational defects in immunological processes may potentially constitute risk factors due to greater susceptibility to infectious diseases. Training using OM-85 (Microbial-derived immunomodulatory) has an effect on crucial immunoregulatory processes have been seen in both pregnant and fetal mice. These results relate to downstream T-regulatory cells, as well as both cDC (Conventional dendritic cell) and pDC (Plasmacytoid dendritic cell) populations. Consequently, these effects lead to increased resistance in mothers and offspring against the inflammatory effects triggered by allergic, viral, and bacterial stimulation (7). The results demonstrate the significance of maternal immunological status and its impact on fetal immune development. However, further research is necessary, especially human clinical studies, to have a more thorough grasp of the underlying immunomodulatory effect from mother to fetus.

Maternal nutritional status has been linked to an increased risk of future disease in offspring. In a cohort study on Chinese adults, it was found that fetal exposure to maternal famine was associated with an increased risk of adulthood MAFLD (metabolic-associated fatty liver disease), especially in women (8). Additionally, prenatal exposure to maternal famine during early gestation was associated with a higher prevalence of coronary heart disease than non-exposed offspring (9). These studies suggested that maternal nutritional status in the various stages of gestation impact on various cardio-metabolic diseases in new born babies and adulthood health conditions.

In addition to pathophysiological conditions, exposure to maternal psychological stress during pregnancy has been identified as a risk of neuropsychiatric disorders in the offspring. A nationwide follow-up cohort study revealed that gender specifically, boys born to mothers who experienced the loss of a child or a spouse during the pregnancy had a higher hazard ratio for attention-deficit hyperactivity disorder (ADHD). These findings indicate severe stress experienced by mothers during pregnancy may increase the risk of ADHD in the male offspring (10). Furthermore, in a population-based cohort study, it was found that severe stress resulting from loss of a close relative during the first trimester of pregnancy was associated with an increased risk of developing schizophrenia in the offspring. These observations lead to the conclusion that the environment may have an influence on neurodevelopment at feto-placental-maternal interface, as suggested by Khashan AS et al. (11). However, the questions regarding how maternal psychological stress influence to fetal physical health is not well understood yet.

Gestational ages and intra-uterine fetal health conditions have been identified as risk factors for various cardio-metabolic diseases in offspring. In a prospective study, a 30-year follow-up period, it was observed that a low gestational age at birth and pre-term birth was associated with elevated systolic blood pressure and insulin resistance in adulthood. Interestingly, these associations were not found to be influenced by birth weight. These findings suggest that length of gestation, rather than fetal growth, may be a major contributor to cardiovascular risk in adulthood (12). Fetal growth restriction (FGR) was associated with future health problems in offspring. According to a study by Sarah J. et al. measured lung function using spirometry in children aged 8-9 years, comparing those with intrauterine growth restriction (IUGR) based on birth weight and a control group. The findings revealed that children with IUGR had reduced lung function compared to the control group (13). A cohort study conducted over a 30-year period explored the association between various birth characteristics and the development of adult fatty liver. Preterm birth, small for gestational age (SGA), low birth weight, and low birth height were found to have significant odds ratios for adult fatty liver. These results indicate that factors determining intra-uterine conditions which reflect immaturity may serve as risk factors for adult fatty liver (14). Offspring whose parents experienced preeclampsia were found to have an increased risk of having a child with a pregnancy complicated by preeclampsia. A studies conducted by Esplin, M.S et al. and Skjaerven, R. et al. demonstrated that the female group’s offspring have higher risk than male group’s. The findings also provide evidence of both paternal and maternal involvement in predisposition to preeclampsia (15, 16). Furthermore, in a population-based cohort study, it was observed that the offspring of mothers who had experienced preterm birth were more likely to develop hypertension and diabetes during their own pregnancies compared to the control group (17). These clinical studies showed that not only gestational ages and intra-uterine conditions in fetus but also maternal obstetric complications have been associated with reproductive complications in offspring.

Many of evidences that maternal inflammation diseases were linked to adverse effects in offspring. The Helsinki Birth Cohort Study (HBCS) found a positive association between maternal BMI and subsequent health outcomes in the offspring, with the strongest association s observed for cardiovascular disease and type 2 diabetes (18). In the Early Childhood Longitudinal Study–Birth Cohort (ECLS–B), a longitudinal study, maternal obesity and overweight were linked to an increased risk of asthma in offspring by the age of 4 (19). In a prospective cohort study that followed children for over 7 years, maternal obesity determined by increased BMI (≥30.0 kg/m^2^) was linked to lower intellectual function in offspring compared to children born to normal range BMI. Furthermore, excessive weight gain during pregnancy was linked to a deficit in IQ in offspring. These findings indicate that maternal obesity and excessive weight gain during pregnancy are associated with lower child IQ (20). From these studies, early prevention of overweight and obesity in women of childbearing age is crucial for intellectual development in offspring. In another cohort study that followed up offspring from the earliest possible age that related to maternal autoimmune diseases were with a higher risk of overall mental disorders in offspring and particularly elevated risk for primary biliary cholangitis. The well-established studies reported that offspring of mothers diagnosed with an autoimmune disease during delivery were at increased risk of developing a wide range of neurodevelopmental disorders (eg, intellectual disability, childhood autism, and ADHD), organic disorders, obsessive-compulsive disorder, schizophrenia, and mood disorders (21). A Danish Birth Cohort Study, which followed up offspring until specific outcomes, found that offspring born to mothers with any diabetes diagnosis during pregnancy were more likely to develop overall psychiatric disorder in offspring compared to unexposed offspring (22). Maternal pregestational diabetes, including type 1 and type 2 diabetes, as well as gestational diabetes were associated with an increased likelihood of developing cardiovascular disease early in life, spanning from childhood to early adulthood (23). Maternal infection during pregnancy has been found to impact negative health outcomes in offspring. In a population-based prospective cohort study, it was observed that offspring whose mother was infected with C. trachomatis during pregnancy were more likely to experience wheezing until the age of 10 years and asthma (24). Furthermore, in a retrospective cohort study conducted by Getahun, D. et al., it was found that fetal exposure to chorioamnionitis, an inflammation of the maternal-fetal interface, combined with preterm delivery, was associated with an increased risk of childhood asthma (25). The offspring exposed to maternal inflammatory health conditions impact on their intellectual growth and cardiovascular health. However, those various large clinical studies are lack of in-depth biological, genetic and molecular mechanisms.

## Maternal obesity and immune cell reprogramming

Children born to obese mothers have a higher risk of experiencing dysregulation in both innate and acquired immunity and an alteration of immune profiling. Umbilical cord blood (UCB) obtained from babies born to overweight and obese mothers showed lower white blood cell counts, primarily attributed to reduced eosinophil counts and a slight reduction in basophil counts compared to babies born to lean mothers. Additionally, UCB from babies born to obese mothers exhibited reduced numbers of total and naïve CD4 T-cells (26). In a study aiming at cord blood stem cell transplantation, UCB obtained from mothers who were obese displayed increased numbers of various lymphocyte subsets, including CD3+, CD4+, CD8+, NKT, and CD8+CD25+Foxp3+ regulatory T (Treg) cells. However, the number of CD24+ cells that are associated with inflammation induced by obesity in adipose tissue was decreased in comparison to the normal-weight group (27). These results emphasize that maternal obesity can influence the immune profiles of newborns, leading to alterations in different immune cell populations that lead to inflammatory conditions.

The link between obesity-induced inflammation and the observed immune changes highlights the importance of understanding the impact of maternal obesity on immune development in offspring. Cifuentes-Zúñigae et al. analyzed monocytes isolated from cord blood from newborns of obese and lean mothers to identify changes in inflammatory and anti-inflammatory cytokines in macrophages. The basal mRNA expression of pro-inflammatory cytokines such as IL-1β and IL-12B and anti-inflammatory cytokine IL-10 were downregulated in the monocytes of children born to obese mothers, while mRNA levels for TNFα in macrophages from children of obese mothers were upregulated compared to non-obese mothers. Furthermore, in response to M2 stimulation, macrophages from obese mothers exhibited further suppression of the expression of the anti-inflammatory mediator IL-10. These changes in IL-1β and IL-10 in monocytes from obese mothers were associated with alterations in DNA methylation patterns in the promoter regions of fetal monocytes. These alterations have long-term implications for the systemic inflammatory balance and are implicated in the development of chronic diseases such as obesity and type 2 diabetes. Moreover, the observed changes in DNA methylation of key inflammatory genes in neonatal monocytes indicate potential *in-utero* programming of immune function induced by maternal obesity (28).

A recent meta-analysis was carried out in the Pregnancy and Childhood Epigenetics (PACE) academy to look into the relationship between methylation of DNA and maternal Gestational Diabetes Mellitus (GDM) in cord blood. The finding reveals that lower levels of methylation in two specific regions of cord blood DNA. The OR2L13 gene promoter, which has been connected to autism spectrum disorders is the first area. The second region is the gene body of the CYP2E1, a gene known to be elevated in diabetes of both type 1 and type 2 (29). According to these data, epigenetic pathways may influence the association between the maternal GDM of autistic spectrum disorders and diabetes-related diseases in offspring.

## Experimental models of the maternal-to-fetal remodeling

Experimental models of maternal-to-fetal metabolic reprogramming have limitations in reproducing the conditions that are generated in pregnant women transcend to offspring. Because the experiment should be designed from one generation to the next generation. For this reason, there is a lack of *in vitro* and *in vivo* models that mimic human models. The diet challenge model conducted in rodents has provided evidence that early exposure to maternal obesity can result in physiological changes and increase the susceptibility of offspring to obesity, hypercholesterolemia, and hypertension (30). Furthermore, experiments using mice fed a high-fat, high-glucose diet, which mimics an obesity-inducing diet that falls within the range of a typical human diet, have demonstrated that exposure to the effects of maternal obesity during developing leads to obesity, as well as cardiovascular and metabolic dysfunction in offspring (31). In a non-human primate model, researchers investigated the effect of maternal consumption of a high-fat/high-calorie diet during pregnancy and the early postnatal period. Offspring born to mothers on the high-fat diet exhibited endothelial dysfunction, characterized by increased expression of vascular inflammatory factors, including VEGF, TNF-α, and ICAM-1. Additionally, their expression levels of several markers associated with fibrinolysis, such as PAI-1 and t-PA, were altered compared to offspring born to mothers on the control diet. Moreover, the offspring from the high-fat diet group displayed thickened intima-media walls and abnormal vessel morphology. These findings suggest that maternal exposure to a high-fat diet may lead to impaired endothelial function in their offspring (32). The study observed that maternal chronic photoperiod shifting (CPS) disrupted amounts of melatonin by Natalia Mendez et al.; it had long-lasting consequences on cardiovascular health and metabolic of adult male offspring in rats. This suggests that alterations in melatonin levels may be crucial in the prenatal programming of chronic diseases associated with gestational chronodisruption. The administration of melatonin medication to mothers could counteract or prevent the alterations shown in adult offspring caused by CPS. Additionally, an inconsistent circadian melatonin rhythm during pregnancy may act as a determining reason for fetal reprogramming for adult female offspring (33). These findings highlight the significance of reprogramming of melatonin and circadian rhythm regulation in prenatal development and the potential implications for long-term health outcomes.

A study using Cohen diabetic rat model for diabetes during pregnancy performed by Sophie Petropoulos et al. demonstrated that gestational diabetes leads to modifications in the placenta’s DNA methylation. This study showed a connection between changes in DNA methylation and pregnant diabetes in genome-wide promoters as well as potential diabetes genes. Importantly, this study not only confirms that gestational diabetes mellitus (GDM) in humans and pregnancy-related diabetes in rats induce changes in the methylome of both rat offspring’s liver and placenta but also highlights that these changes affect cardiovascular and metabolic disease have comparable functional mechanisms. Furthermore, several genes were found to be shared between both species, further supporting the similarities between humans and the Cohen diabetic rat model in relation to diabetes-related alterations in DNA methylation (34).

In a rat model of a pre-eclampsia-like syndrome treated by low-dose lipopolysaccharide (LPS) during pregnancy, female offspring born to F0 mother displayed mild systolic dysfunction, increased expression of genes associated with cardiac growth, altered glucose tolerance, and coagulopathy. On the other hand, male offspring demonstrated changes in glucose tolerance and an accumulation of visceral fat. F2 offspring from F1 females born to LPS-treated F0 mothers experienced growth restriction, which was correlated with CD68 positivity, indicating the presence of macrophages, and reduced expression of glucose transporter-1 in their uteroplacental units (35). These findings suggest that maternal inflammation may serve as a risk factor for cardiovascular and metabolic disease in the offspring generation after generation. Furthermore, the impact of inflammation appears to transcend generations, as evidenced by the observed effects on F2 offspring, highlighting the potential intergenerational influence of abnormal maternal inflammation on the health outcomes of subsequent generations.

## Maternal-to-fetal reprogramming of umbilical cord blood

Umbilical cord blood monocytes derived from babies born to obese mothers exhibit impaired responsiveness to Toll-like receptor 1/2 (TLR1/2) and TLR4 ligands, which are pattern recognition receptors involved in recognizing pathogens such as *L. monocytogenes*, *M. hominis*, *Enterobacteriaceae*, *C. albicans*, *Cytomegalovirus* (CMV), and respiratory syncytial virus (RSV). This compromised response increases the susceptibility of these newborns to neonatal infections. RNA-sequenced data obtained from UCB monocytes of infants born to obese mothers revealed reduced responses to LPS stimulation. This diminished response can be attributed to a significant decrease in the number of transcription factors that regulate genes activated by the metabolic status of monocytes (36). Furthermore, babies born to obese mothers exhibit differential regulation of gene function. Analysis of gene expression using fetal cord blood RNA extracted from obese mothers demonstrated significant activation of upstream regulators involved in inflammatory signaling. Additionally, factors related to insulin receptor signaling, lipid homeostasis, and cellular responses to oxidative stress were also found to be dysregulated (37). These findings suggest that maternal obesity has an impact on fetal gene expression during pregnancy, leading to dysregulation of inflammatory and immune signaling pathways, as well as increased oxidative stress.

## *In vivo* model

In a mouse model conducted by Friedman et al., it was discovered that offspring born to obese mothers exhibited accelerated fibrosis in the liver. This accelerated fibrosis was accompanied by early recruitment of inflammatory macrophages and microbial heterogeneity during early life. The researchers also observed that bone marrow-derived macrophages (BMDM) played a role in polarizing the inflammatory state during early life and promoting the progression of both inflammatory and fibrotic phases in the offspring. Importantly, these effects persisted into adulthood (38). This study highlighted the detrimental consequences of maternal obesity on liver health in offspring, demonstrating an accelerated fibrotic response accompanied by early inflammatory macrophage recruitment and microbial heterogeneity.

In a germ-free mouse model, Soderbog et al. conducted a study comparing the fecal microbiota of 2-week-old pups born to obese mothers versus normal-weight mothers. The researchers discovered that the fecal microbiome of offspring born to obese mothers showed histological patterns similar to those observed in pediatric non-alcoholic fatty liver disease, due to dysbiosis. Additionally, they observed increased intestinal permeability, impaired macrophage phagocytosis, and reduced cytokine production in the offspring born to obese mothers, suggesting dysfunction in macrophage activity. Furthermore, when the offspring born to obese mothers were exposed to a Western diet, they experienced excessive weight gain and developed non-alcoholic fatty liver disease (39). These findings suggest that the combination of maternal obesity and dysbiosis in early life contributes to the development of metabolic disorders, including non-alcoholic fatty liver disease, in the offspring.

In a mouse model study conducted by Edlow et al., brain microglia and CD11b+ placental cells were isolated from fetuses born to mothers who were fed a high-fat diet to induce obesity. The researchers found a strong correlation between the production of proinflammatory cytokines and the presence of brain microglia in the offspring mice exposed to a high-fat diet. CD11b+ cells obtained from fetuses exposed to maternal obesity exhibited an excessive response to LPS and increased production of TNF-α in both the placenta and brain, as compared to the control group. These findings revealed the impact of maternal obesity on fetal brain microglia and placental macrophages. Maternal obesity was found to trigger excessive production of inflammatory cytokines by these cells in response to immunological stress (40). The study underscores the role of maternal obesity in promoting a proinflammatory environment in both the placenta and the developing brain of the offspring, which may have long-lasting implications for neurodevelopment and immune function.

Restricting calorie intake during pregnancy led to impaired intrauterine development, while calorie restriction has been shown to improve Nrf2 signaling and encourage adult mouse lifespan by reducing reactive oxygen species. The former is associated with fetal reprogramming, elevating the danger of adult diabetes, obesity, and metabolic syndrome. George I. Habeos et al. demonstrated that calories restricted by the mother increased the expression of genes involved in the integrative reaction to stress as well as the fetal liver’s antioxidant and cytoprotective enzymes. These changes are particularly significant as they may be how adult mice respond to various stimuli, including toxins, excessive nutrient intake, and food shortage (41). The identified alterations in metabolic and antioxidant/cytoprotective genes offer insights into the potential mechanisms that the impact of maternal calorie restriction throughout the long term on the offspring’s response to environmental challenges, nutrient imbalances, and limited food availability.

The presence of GDM and intrauterine growth restriction (IUGR) has been found to have an impact on the methylation patterns in offspring. These changes in DNA methylation suggest the involvement of shared genes and pathways that may be connected with a higher chance of getting type 2 diabetes in the future. Particularly, the protein C3orf31 is responsible for assembling and maintaining mitochondrial translocators, which have a known intergenic SNP between it and the VGLL4 gene of interest. Furthermore, C3orf31 is associated with insulin resistance and ACYP2, a gene linked to coronary artery disease (42). These findings suggest that the altered methylation patterns in genes such as C3orf31 and ACYP2 may be a factor in the elevated susceptibility of those with type 2 diabetes exposed to maternal GDM and IUGR. Further research in gene regions and related pathways may offer useful information about the underlying mechanisms linking maternal conditions during pregnancy with the potential occurrence of type 2 diabetes in descendants.

## Epigenetic regulations of maternal-to-fetal reprogramming

The maternal metabolic environment during pregnancy has been linked to fetal epigenetic programming. In the clinical birth cohort study involving 40 mother-infant dyads, it was observed that maternal lipid metabolites, including very long chain fatty acids, medium chain acylcarnitines, and histidine, remained stable from the first trimester to delivery and exhibited significant correlations with cord blood metabolites. Moreover, these metabolites were found to be associated with fetal DNA methylation patterns, suggesting a strictly regulated relationship between maternal lipid metabolites and DNA methylation (43). Furthermore, pregnancy complications were found to be associated with developmental epigenetic aging processes in the fetus. A cohort study that harmonized data from 12 pregnancy cohorts across the United States revealed that prenatal exposure to maternal gestational diabetes and preeclampsia led to decelerated epigenetic aging at birth, particularly in female offspring, compared to unexposed neonates. These findings imply that prenatal exposure to gestational diabetes and preeclampsia delays biological maturity, especially in female offspring. Ladd-Acosta C et al. emphasized the importance of further understanding epigenetic mechanisms involved in fetal programming and the relevance of the intrauterine environment in shaping downstream health outcomes in offspring (44). In offspring from dams fed an HFD (High-fat diet), changes in DNA methylation levels have been observed in the leptin and Ppar- α (Peroxisome proliferator-activated receptor α) promoters. Specifically, the female offspring’s livers can retain the leptin promoter’s DNA methylation state from the oocytes of HFD mothers. This methylation pattern influences the regulation of leptin expression, and lower leptin expression has a connection to weight gain. The livers of female offspring of mothers given a high-fat diet produce more Ppar-mRNA and Ppar-protein, indicating that DNA methylation is crucial for offspring’s fat and overweight (45).

Epidemiological research has shown a correlation between the prevalence of gestational diabetes mellitus or pregnancy-related maternal obesity and increased risk factors for cardiometabolic disorders in offspring. Researchers have identified DNA methylation biomarkers that persist throughout the first year of life, which can distinguish between offspring born to mothers who are overweight and those born to mothers who have gestational diabetes. Notably, enrichment analysis revealed that these alterations in DNA methylation patterns are linked to genes and pathways such as CPT1B, SLC38A4, SLC35F3, and FN3K that are involved in fatty acid metabolism, mitochondrial bioenergetics, and postnatal development. 6 months of offspring’s development appear to be a crucial time for epigenetic remodeling, where these changes occur. The findings suggest that systemic intrauterine programming is associated with pregnancy-related diabetes and obesity, which influences the methylome of children after birth. Metabolic pathways are affected by these epigenetic alterations, and they may interact with the common postnatal development processes (46).

Abalo Chango et al. presented a thorough analysis of the significance of dietary elements in altering the epigenetic processes that control gene expression. The effects of maternal diet elements on histone modification, microRNAs, and DNA methylation are given special attention. The crucial stages of fetal development and the first several months after birth are highlighted as times when food components have a profound influence on metabolic programming and epigenetic processes. The authors emphasized the potential for effective maternal nutrition in adult chronic disease prevention. By comprehending how dietary components impact the operation of the epigenetic machinery and have an impact on the expression of metabolic genes, By understanding how nutritional factors affect the epigenetic mechanisms and influence metabolic gene expression, it can be possible to develop effective strategies to enhance long-term health results (47). The evidence from both epidemiological and experimental proof clearly shows that insufficient pregnant woman’s diet and maternal obesity expose developing offspring to unfavorable intrauterine cues, leading to long-term programming of organs for chronic disease throughout their lives. Both obesity and an HFD during pregnancy have been associated with reproductive issues, embryonic growth limitation, developmental problems with the brain, heart issues, and endocrine malfunction, supported by both epidemiological and experimental evidence. Moreover, the hematological system’s vulnerability to metabolic imbalance throughout development is highlighted by the relationship between pregnant woman’s diet, child health, and maternal health that involve immune offspring derived from the hematopoietic stem cell (HSC) compartment (48).

Elevated concentrations of non-esterified fatty acids (NEFA) during the maturation of oocytes *in vitro* or embryonic growth have been found to impact the transcriptome and epigenetic profiles of resulting blastocysts. This effect is particularly observed in females with metabolic diseases characterized by increased lipolysis. Interestingly, early cleavage embryos exhibited a higher degree of epigenetic dysregulation compared to oocytes during maturation, making them especially more susceptible to change *in vitro* conditions. These results imply that abnormal maternal metabolism can disrupt the normal epigenetic reprogramming of embryos, potentially having long-term consequences on growth and health in later years of adulthood (49). Individuals exposed to gestational diabetes during gestation have an increased likelihood of later life having metabolic and cardiovascular diseases. This association can be attributed, at least in part, with relation to controlling gene expression through epigenetic mechanisms. Specifically, methylation patterns at certain CpG sites associated with intrauterine exposure have been linked to reduced insulin secretory function, higher body mass index (BMI), and a higher chance of developing type 2 diabetes later. These specific methylation profiles can influence insulin secretion, contribute to weight gain, and increase susceptibility to type 2 diabetes (50).

Pregnant women with gestational diabetes mellitus (GDM) have a higher chance of having children who have metabolic diseases; this risk may be mediated by epigenetic mechanisms. To gain a comprehensive understanding of the complicated interaction between pre-pregnancy maternal BMI and GDM in both epigenetic and metabolic health of offspring, further longitudinal studies incorporating extensive clinical information and biospecimens are needed. There is a chance that changes to the methylation patterns of flowing blood cells will mean the underlying processes of diabetes-related target organ damage, and serve as useful indicators for predicting metabolic disease in the offspring of mothers with GDM (51). Pre-conception obesity has a connection to impaired sex-specific cardiometabolic health in offspring. Specifically, in female offspring, maternal obesity before pregnancy was linked to changes 876 CpG sites, while in male offspring, it was linked to changes in 293 CpG sites. Among these, 57 CpG sites, along with the top 18, were found to be associated with the TAPBP gene in female progeny. Furthermore, Males also showed variations in CpG methylation at the TAPBP gene. These findings suggest the presence of sex-specific effects that may contribute to the observed effects of maternal obesity on particular sex (52). Maternal BMI was discovered to be connected to methylation variations at 9,044 different genomic sites in infants. However, adjusting for projected cell proportions, the amount of relevant CpGs was decreased to 104, with 86 sites overlapping with the unadjusted model. In both infants and adolescents, the direction of the association remained consistent at 72 out of 86 shared locations, suggesting persistent signals overtime. Only 8 out of 86 sites showed evidence of an effect of the mother’s BMI during pregnancy on the offspring’s methylation. However, rather than a causal intrauterine mechanism, these impacts were probably impacted by lifestyle or genetic variables. In conclusion, this well-powered research showed strong relationships between variations in a mother’s obesity and newborn blood DNA methylation (53).

## Conclusion

In summary, the studies in this review highlight the various spectrum of disorders related to mothers and their impacts on the offspring (**Table 1**). The mechanisms were related to the effect of maternal obesity and gestational diabetes on offspring through epigenetic mechanisms. The identified DNA methylation biomarkers provide insights into the underlying molecular pathways involved in fatty acid metabolism, postnatal development, and mitochondrial bioenergetics. These findings contribute to our understanding of the interplay between prenatal factors, epigenetic regulation, and the development of cardiometabolic disorders in children. Lodge-Tulloch NA et al. suggested a potential mechanism linking the acquisition of trained immunity after exposure to abnormal maternal inflammation to increased disease risk (54). A model that may better explain this reprogramming mechanism is proposed to look at immune memory in the bone marrow by analyzing bone marrow cells from HFD mouse mothers and their fetuses. Specifically, by inducing obesity in mothers in a mouse model, causing low-intensity chronic inflammation that leads to the formation of immune memory in the bone marrow, and then providing a secondary stimulus by feeding their offspring a high-fat diet after birth, we can study the epigenetic regulatory mechanisms that influence the development of obesity. Overall, this review underscores the significant impact of maternal diet on epigenetic processes and metabolic programming (**Table 2**). It highlights the potential of nutritional interventions during critical development periods to positively influence the epigenetic regulations of genes associated with chronic diseases in adulthood. However, the linking mechanism studies *in vitro* and *in vivo* were very limited. This comprehensive review sheds light on the intricate relationship between maternal metabolic health, early-life exposures, immune reprogramming, and the subsequent development of obesity and cardiovascular diseases in offsprings born to such mothers. The knowledge synthesized here will serve as a foundation for further research endeavors aimed at elucidating the complex mechanisms driving these associations. Gaining a deeper understanding of the epigenetic mechanisms involved in the developmental origins of obesity and cardiovascular disease will open new avenues for innovative preventive and therapeutic strategies, thus mitigating the long-term health consequences associated with maternal obesity and gestational diabetes.

**Table 1:** Summarize the effects on the fetus depending on the maternal disease condition.

Maternal conditions	Diseases	Influences on the fetus	Study types	Ref
Famine		Metabolic Associated Fatty Liver Disease (MAFLD)	Clinical study	(8)
		Coronary heart disease	Clinical study	(9)
Psychological stress		Attention-deficit hyperactivity disorder (ADHD)	Clinical study	(10)
		Schizophrenia	Clinical study	(11)
Pregnancy complications	Preterm	Cardiovascular risk	Clinical study	(12)
		Fatty liver	Clinical study	(14)
		Pregnancy complications	Clinical study	(17)
	Intrauterine growth retardation (IUGR)	Poorer lung function	Clinical study	(13)
	Preeclampsia (PE)	Preeclampsia (PE)	Clinical study	(15) (16)
	Gestational Diabetes Mellitus (GDM), Preeclampsia (PE)		Clinical study	(29)
			Rat model	(34)
			Clinical study	(42)
		Reduced biological maturity	Clinical study	(44)
			Clinical study	(46)
			Clinical study	(50)
			Human study	(51)
Inflammation (Inflammation associated disease)		Cardiometabolic alterations, Intrauterine growth restriction (IUGR)	Rat model	(35)
	Overweight	Adverse cardio-metabolic profile in offspring	Clinical study	(2, 3)
		Obesity	Clinical study	(4)
		Cardiovascular disease, Type 2 diabetes	Clinical study	(18)
		Asthma	Clinical study	(19)
		Endothelial dysfunction, Increased expression of vascular inflammatory factors	NHP(Primate model)	(32)
			Mouse model	(45)
	Obesity	Asthma	Clinical study	(5) (6)
		Lower child IQ	Clinical study	(20)
		Metabolic disease (hypertension,fatty liver)	Mouse model	(30)
		Obesity/cardiovascular and metabolic dysfunction	Mouse model	(31)
			Clinical study	(52)
			Clinical study	(53)
	Autoimmune diseases	Mental disorders	Clinical study	(21)
	Diabetes	Psychiatric disorder	Clinical study	(22)
		Cardiovascular disease	Clinical study	(23)
	Chlamydia trachomatis	Asthma	Clinical study	(24)
	Chorioamnionitis	Asthma	Clinical study	(25)

**Table 2:** Summarize the epigenetic impact of maternal disease on the fetus.

Potential Targets	Causes	Effects	Study Types	Ref
Myeloid cell reprogramming	Microbial-derived immunomodulator OM-85	Increased resistance in mothers and offspring both against the inflammatory effects triggered by allergic, viral and bacterial stimulation	Mouse model	(7)
	Obesity	Programming of the neonatal immune system and decrese CD4+ t cell and eosinophils in cord blood	Clinical study	(26)
		Increase in natural killer T cells and CD8+ regulatory T cells in cord blood units	Clinical study	(27)
		Exposure to a maternal WD induces early gut dysbiosis and disrupts intestinal tight junctions, resulting in BMDM polarization and induction of proinflammatory and profibrotic programs in the offspring that persist into adulthood.	Mouse model	(38)
		Inf-ObMB mice show increased intestinal permeability, reduced macrophage phagocytosis, and dampened cytokine production suggestive of impaired macrophage function.	Mouse model	(39)
		Aternal obesity primes both placental macrophages and fetal brain microglia to overproduce a proinflammatory cytokine in response to immune challenge.	Mouse model	(40)
DNA methylation	Gestational Diabetes Mellitus(GDM)	Epigenetic pathways that influence the association between the risk and maternal GDM of autistic spectrum disorders and diabetes-related diseases in offspring.	Clinical study	(29)
		Changes in the methylome of both rat offspring’s liver and placenta but also highlights that these changes affect cardiovascular and metabolic disease.	Rat model	(34)
		Methylation patterns at certain CpG sites associated with intrauterine exposure have been linked to reduced insulin secretory function, higher body mass index, and a higher chance of developing type 2 diabetes later.	Clinical study	(50)
	Gestational Diabetes Mellitus(GDM), Preeclampsia(PE)	Decelerated gestational epigenetic age	Clinical study	(44)
	Gestational Diabetes Mellitus(GDM), Intrauterine growth restriction(IUGR)	Altered methylation patterns in genes be a factor in the elevated susceptibility to those with type 2 diabetes exposed to maternal GDM and IUGR	Clinical study	(42)
	Gestational Diabetes Mellitus(GDM), Pregnancy related maternal obesity	Systemic intrauterine programming is associated with pregnancy-related diabetes and obesity which influences the methylome of children after birth.	Clinical study	(46)
	Obesity	Changes in DNA methylation of key inflammatory genes in neonatal monocytes	Clinical study	(28)
		Maternal Pregravid Obesity Remodels the DNA Methylation Landscape of Cord Blood Monocytes Disrupting Their Inflammatory Program	Clinical study	(36)
		Maternal obesity affects fetal gene expression at term, implicating dysregulated brain development, inflammatory and immune signaling, glucose and lipid homeostasis, and oxidative stress	Clinical study	(37)
		Presence of sex-specific effects that may contribute to the observed effects of maternal obesity on particular sex.	Clinical study	(52)
		Maternal BMI was discovered to be connected to methylation variations at different genomic sites in infants.	Clinical study	(53)
	Maternal lipid metabolites	The correlation between maternal and fetal metabolites influences DNA methylation, potentially impacting overall health.	Clinical study	(43)
	High-fat Diet(HFD)	Methylation pattern influences the regulation of leptin expression and lower leptin expression has a connection to weight gain.	Mouse model	(45)
Others	Maternal Chronic Photoperiod Shifting(CPS)	CPS disrupts amounts of melatonin and have long-lasting consequences on cardiovascular health and metabolic of adult male offspinrg in rats.	Rat model	(33)
	Pregnant woman’s diet	Calorie restriction during pregnancy induces transcription of cytoprotective/antioxidant genes in the fetal liver.	Mouse model	(41)
		Insufficient pregnant woman’s diet and maternal obesity leads to long-term programming of organs for chronic disease throughout lives. Both obesity and HFD during pregnancy have been associated with embryonic growth limitation, developmental problems with the brain, heart isssue and endocrine malfunction.	Mouse model	(48)
	Non-esterified Fatty Acids(NEFA)	Abnormal maternal metabolism can disrupt the normal epigenetic reprogramming of embryos, potentially having long-term consequences on growth and health in later years of adulthood.	Human study	(49)
	Gestational Diabetes Mellitus(GDM)	Pregnant women with gestational diabetes mellitus have a higher chance of having children who have metabolic diseases that risk may be mediated by epigenetic mechanisms.	Human study	(51)

## Author contributions

YA: Conceptualization, Funding acquisition, Investigation, Resources, Supervision, Writing – original draft, Writing – review & editing. HK: Conceptualization, Investigation, Writing – review & editing. JK: Conceptualization, Writing – original draft, Writing – review & editing, Resources, Visualization. HM: Conceptualization, Writing – original draft, Writing – review & editing, Resources, Visualization. MC: Writing – original draft, Writing – review & editing, Conceptualization, Resources, Visualization.
